# Comparative transcriptome analysis revealed omnivorous adaptation of the small intestine of Melinae

**DOI:** 10.1038/s41598-021-98561-0

**Published:** 2021-09-27

**Authors:** Lidong Wang, Xiufeng Yang, Shengyang Zhou, Tianshu Lyu, Lupeng Shi, Yuehuan Dong, Honghai Zhang

**Affiliations:** Qufu Normal University, Qufu, 273165 China

**Keywords:** Molecular ecology, Molecular evolution

## Abstract

As the main digestive organ, the small intestine plays a vital role in the digestion of animals. At present, most of the research on animal feeding habits focuses on carnivores and herbivores. However, the mechanism of feeding and digestion in omnivores remains unclear. This study aims to reveal the molecular basis of the omnivorous adaptive evolution of Melinae by comparing the transcriptome of the small intestines of Asian Badgers (*Meles leucurus*) and Northern Hog Badgers (*Arctonyx albogularis*). We obtained high-quality small intestinal transcriptome data from these two species. Key genes and signalling pathways were analysed through Gene Ontology (GO), Kyoto Encyclopedia of Genes and Genomes (KEGG) and other databases. Research has mainly found that orthologous genes related to six enzymes have undergone adaptive evolution. In addition, the study also found three digestion-related pathways (cGMP-PKG, cAMP, and Hippo). They are related to the digestion and absorption of nutrients, the secretion of intestinal fluids, and the transport of food through the small intestine, which may help omnivorous animals adapt to an omnivorous diet. Our study provides insight into the adaptation of Melinae to omnivores and affords a valuable transcriptome resource for future research.

## Introduction

Diet is the most powerful selection factor for animals^1^. Each lineage of placental mammals has its own unique diet, but we can still divide them into herbivores, omnivores, and carnivores based on food types^2^. Mustelidae is a special group in the Carnivore order, whose members are mostly made up of obligate carnivores, so they are also known as hypercarnivores^3^. However, there are also omnivorous species in the Mustelidae, such as Asian Badge and Northern Hog Badger, Honey badgers (*Mellivora capensis*), and Wolverines (*Gulo gulo*). It is common in mammals for species in the same family to have different diets. For example, polar bears (carnivores), grizzly (omnivores), and panda (herbivores) bears in Ursidae^4,5^; and foxes (omnivores) and wolfs (carnivores) in Canidae. Carnivorous species of the Mustelidae are relatively slender and flexible, while omnivorous species are slightly bloated. Diversities in diet conditions cause many animals to undergo adaptive evolution in physiology, biochemistry and morphology. In previous studies of carnivores, researchers have identified a number of key physiological and biochemical characteristics related to diet, such as differences in the concentration of digestive enzymes^6^, shortening of the digestive tract^7^, and amino acid requirements, and changes in genes related to taste^8,9^. However, relatively little research has been done on omnivorous animals.

Mustelidae is one of the most widely distributed carnivores and thrives in a variety of environments. Asian Badgers and Northern Hog Badgers are of similar size. The most obvious difference between the two species is that Asian Badgers have noses that are similar to dogs and Northern Hog Badgers have noses that are similar to pigs. At present, studies on Asian Badgers and Northern Hog Badgers mainly focus on feeding habits, anatomy, biomedicine, and population dynamics. Ye studied the eating habits of Asian Badgers in Eurasia from different aspects and found that animal food accounted for 60% and plant food accounted for 40% of the composition of the food. They also compared the feeding habits of Asian Badgers with different geographical distributions and found that Asian Badgers are euryphagous animals. Two main factors affect the variety of Asian Badgers' food, namely, the availability of food and the abundance of resources^10^. Zhou investigated the seasonal dietary changes and food resource changes of the Northern Hog Badger in a subtropical forest and found that it mainly feeds on earthworms and fruits supplemented by arthropods, invertebrates, and a small number of vertebrates (such as birds, small reptiles, and small mammals)^11^. However, most of these studies have focused on phenotypes and have involved a few molecular adaptation studies.

While most members of the Mustelidae are carnivores, Asian Badgers and Northern Hog Badgers are omnivorous. Studies on the digestion of Melinae species can provide a basis for the evolution of feeding habits of the entire Mustelidae. At the same time, humans are omnivorous animals, so the study on Melinae digestion can provide a reference for human health. In this study, transcriptome sequencing was performed on the small intestine of three Asian Badgers and three Northern Hog Badgers. Selective stress analysis and enrichment analysis identified some genes that may be subject to selective stress and are associated with intestinal digestion and absorption. These data provide a valuable transcriptome resource for future research on omnivorous animals and are potentially valuable in the face of the current effects of biodiversity loss. In addition, our findings provide data to further understand the adaptation of other species and humans to food diversity.

## Results

### Summary of sequencing data

**Figure 1:**
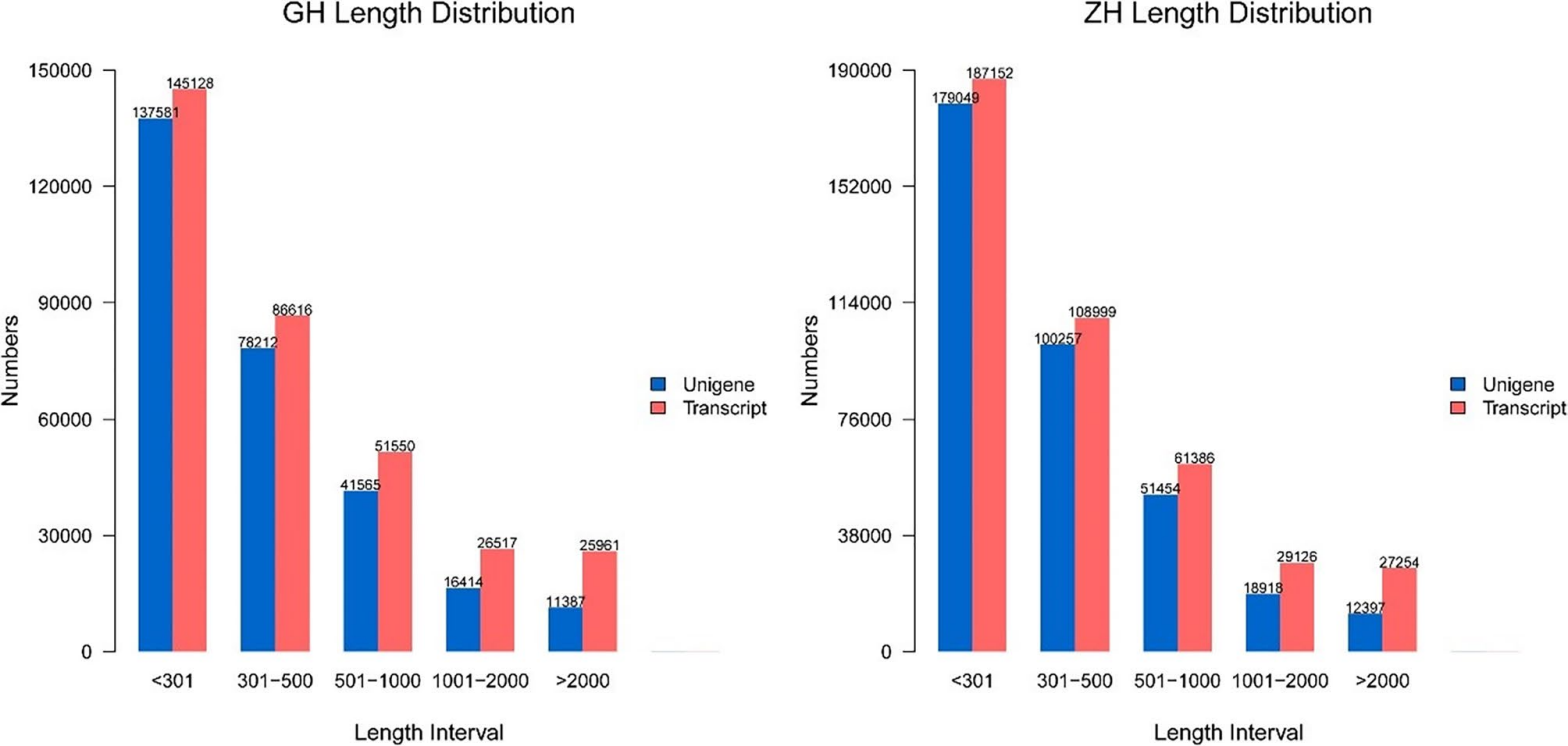
Length and quantity distribution of transcripts and unigenes.

We obtained 168 million and 180 million 250 bp reads from Asian Badger and Northern Hog Badger, respectively. After removing transcripts and unigenes below 200 bp, we obtained 335,772 transcripts and 285,159 unigenes belonging to Asian Badger and 413,917 transcripts and 362,075 unigenes belonging to Northern Hog Badger (Table 1). Next, we analysed the length distribution of the unigenes and transcripts in these two species (Fig. 1). Their N50 of transcript length is longer than 1000 bp, and their N50 of unigene length is longer than 600 bp. The average GC content of the transcriptome data of Asian Badge was 52.71%, a value slightly higher than that of the Northern Hog Badger, which was 52.12% (Table 1).

**Table 1:** Summary of the transcriptome of Asian Badgers and Northern Hog Badgers.

	GH	ZH
Clean reads	153,316,230	164,335,646
Clean bases(Gbp)	23	24.65
Total number of transcripts	247,242	441,251
Total number of unigenes	214,119	384,783
Average GC content (%)	52.71	52.12
N50 of transcript	1081	1268
N50 of unigene	658	645

### Functional annotation and classification of the assembled unigenes

**Figure 2:**
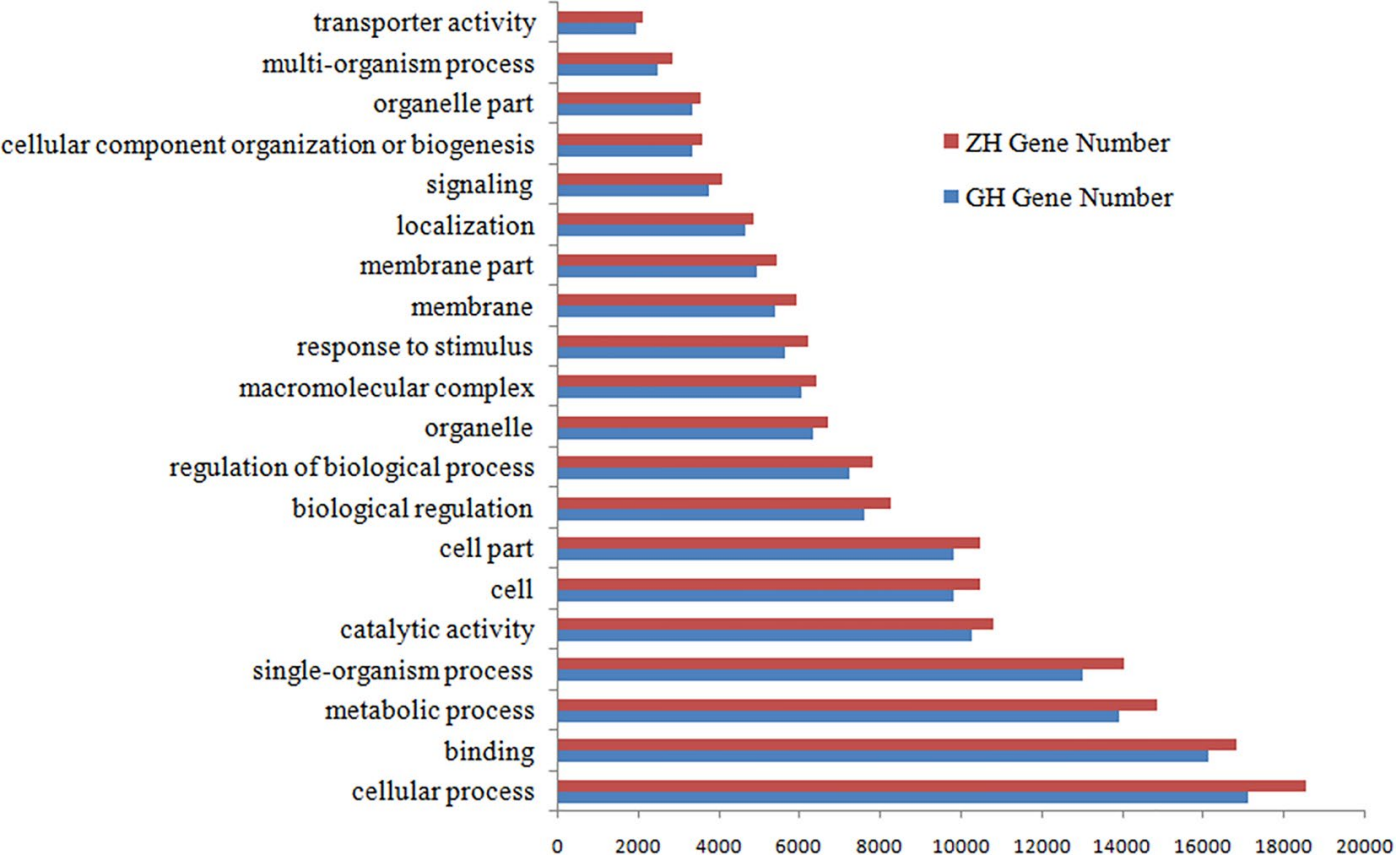
GO term Top20 for GH and ZH.

The success rate of annotation of these research data in the seven databases is shown in Table 2. In total, 34,150 (ZH) and 31,632 (GH) unigenes had GO terms (Table 2). Among them, there were three GO items related to digestion: positive regulation of the digestive system process (GH and ZH both have one gene), digestive tract development (GH and ZH both have four genes), and digestion (GH has five genes, ZH has three). Next, we compared the GO terms of Asian Badger and Northern Hog Badger transcriptomes and found that the distributions pattern of gene functions from these two species were particularly similar (Fig. 2). This predictable result indicates that there is no bias in the construction of the libraries from the Asian Badger and Northern Hog Badger. For both species, in the three main partitions (cellular component, molecular function, and biological process) of the GO classification, ‘Cellular process’, ‘Binding’ and ‘Metabolic process’, terms were principal individually (Fig. 2). In total, 8915 (ZH) and 10,203 (GH) unigenes had KOG terms (Table 2). In addition, 15,667 (ZH) and 17,823 (GH) were mapped to the Kyoto Encyclopedia of Genes and Genomes (KEGG) pathways (Table 2) and grouped into 32 subclasses. Interestingly, the digestive system subcategory contains 695 and 611 unigenes in Asian Badger and Northern Hog Badger, respectively, involving 9 pathways, namely, bile, gastric acid, pancreatic, salivary secretion, carbohydrate, protein, vitamin, fat digestion and absorption, and mineral absorption.

**Table 2:** Gene annotation success rate statistics.

Annotation database	ZH's unigene nums	GH's unigene nums
NR	27,062 (7.47)	34,701 (12.16)
NT	118,618 (32.76)	101,349 (35.54)
KEGG	15,667 (4.32)	17,823 (6.25)
SwissProt	27,702 (7.65)	28,345 (9.94)
PFAM	34,049 (9.4)	31,487 (11.04)
GO	34,150 (9.43)	31,632 (11.09)
KOG	8915 (2.46)	10,203 (3.57)
Annotated in all Databases	4407 (1.21)	6391 (2.24)
Annotated in at least one Database	131,381 (36.28)	112,596 (39.48)
Total Unigenes	362,075 (100)	285,159 (100)

Annotation in NR, NT, KO, SwissProt, PFAM, GO, and KOG: the number and proportion of unigenes successfully annotated in seven databases.

Annotated in all databases: the number and percentage of unigenes successfully annotated in all seven databases.

Annotated in at least one database: the number and percentage of unigenes successfully annotated with at least one database.

### Analysis of orthologous genes

The transcriptome evolution of different species can be understood by comparing transcriptome data. We analysed the possible orthologous genes between the transcriptome of Asian Badger and Northern Hog Badger obtained in this study. We selected a total of 5227 homologous gene pairs from these four species. After 5227 pairs of homologous genes were optimized and screened, 943 orthologous gene pairs were obtained (Supplementary Table S1).

**Figure 3:**
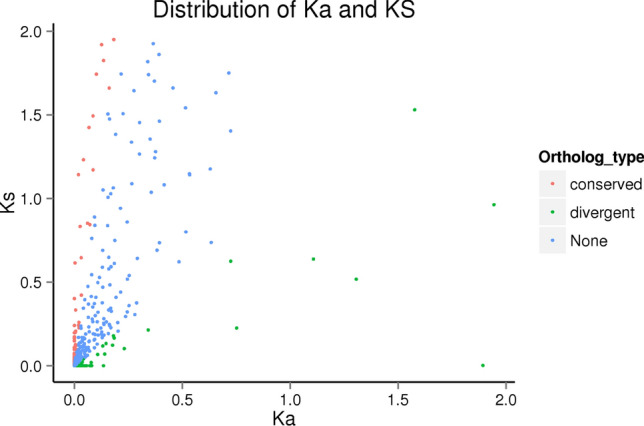
Orthologous distribution of Ka and Ks distribution.

To explore whether the genes related to small intestinal digestion in Asian Badger and Northern Hog Badger have undergone adaptive evolution. We can predict the genes that affect the evolution of the two species through selection pressure on orthologous genes^12^. We selected 473 orthologous gene pairs with Ka/Ks > 1 called divergent orthologous genes from the Ka/ks analysis results. We obtained 1263 orthologous gene pairs with Ka/Ks < 0.1, which were called conserved orthologous genes (Fig. 3). These genes are relatively conserved, and they are subject to strong selection constraints in evolution.

### GO enrichment analysis of divergent and conserved orthologous genes

After screening out divergent and conserved orthologous genes, studying the distribution of divergent and conserved orthologous genes in Gene Ontology will clarify the manifestation of evolutionary differences in species sequences on gene function. Of the 473 divergent orthologous gene pairs identified, 260 were enriched in 1510 GO terms, 920 were biological processes, 370 were molecular functions and 220 were cellular components (Supplementary Table S2). A significant analysis of GO enrichment results (p value < 00.05) showed that 43 GO terms were significantly enriched. As shown in Table 3, 8 terms were related to the activities of various enzymes [steroid dehydrogenase activity (GO: 0016229), nuclease activity (GO: 0004518), plastocyanin reductase activity (GO: 0009496), oxidoreductase activity (GO: 0052880), *N*-acetyltransferase activity (GO: 0008080), and endonuclease activity (GO: 0004519)].

**Table 3:** Part of the results of the GO enrichment analysis.

GO ID	Enzymes	Type	p value
GO:0003854	Steroid dehydrogenase	MF	0.026252
GO:0016229		MF	0.026252
GO:0033764		MF	0.026252
GO:0004518	Nuclease	MF	0.02771
GO:0009496	Plastoquinol-plastocyanin reductase	MF	0.039335
GO:0052880	Oxidoreductase	MF	0.039335
GO:0008080	*N*-Acetyltransferase	MF	0.040944
GO:0004519	Endonuclease	MF	0.045448

### KEGG enrichment analysis of divergent orthologous genes

**Figure 4:**
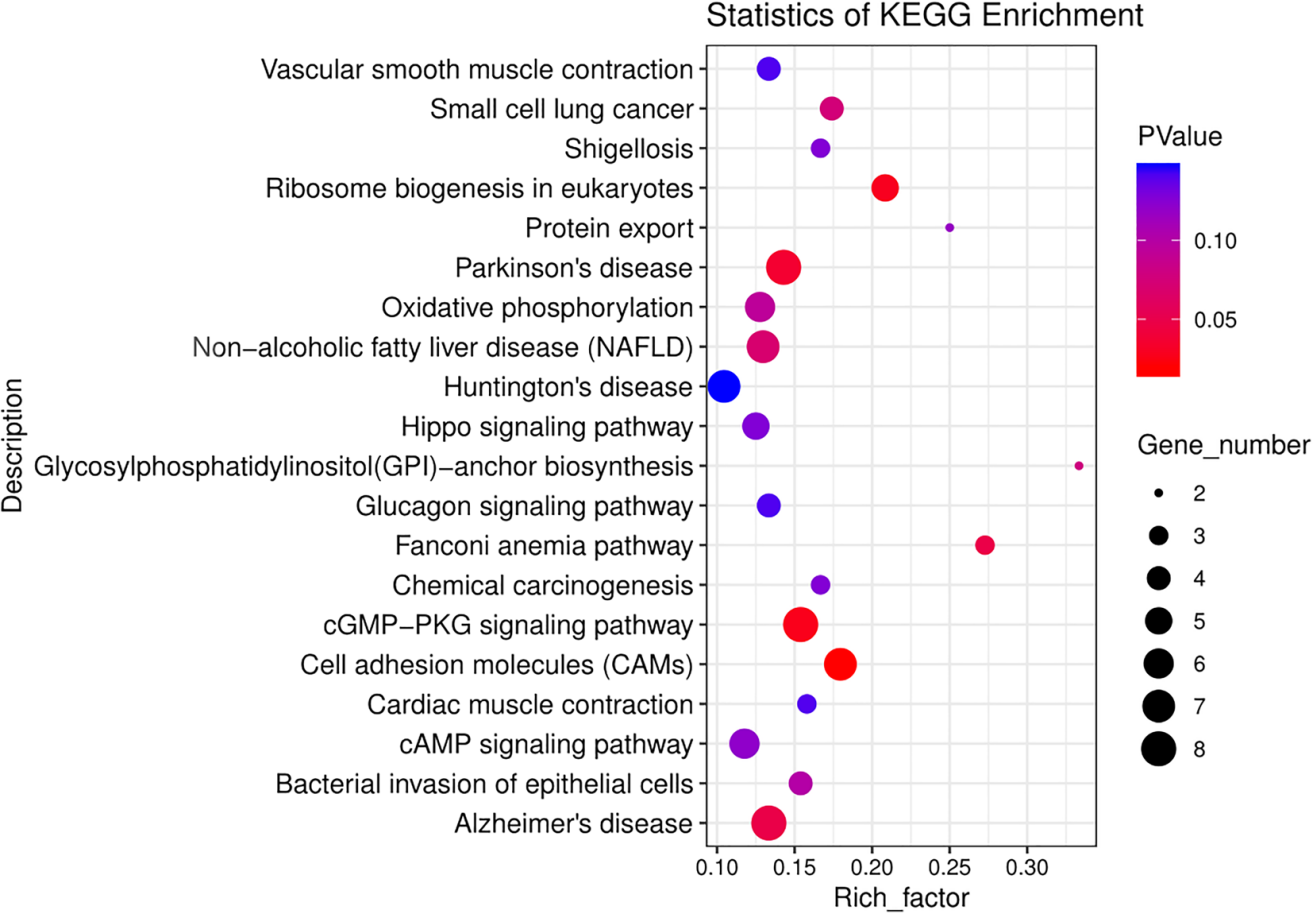
KEGG pathway enrichment scatter plot.

In organisms, different genes coordinate with each other to perform their biological functions. Through significant enrichment of pathways, the most important biochemical metabolic pathways and signal transduction pathways involved in divergent or conserved genes can be determined. Our enrichment analysis of 473 pairs of divergent orthologous genes showed that 117 pairs of divergent orthologous genes were enriched in 195 KEGG pathways, and the number of genes enriched in each pathway was between 1 and 9 (Supplementary Table S3). The 20 most significant pathways are shown in Fig. 4. Among these KEGG pathways of the top 20 significance, 6 were significantly enriched (p value < 0.05), and the most significant enrichment was the cell adhesion molecule pathway, which enriched 7 divergent orthologous genes, followed by the cGMP-PKG signalling pathway enriched in 8 genes, ribosomal synthesis in eukaryotes enriched in 5 genes, Parkinson’s disease enriched in 8 genes, Fanconi anaemia pathway enriched in 3 genes and Alzheimer's disease pathway enriched in 8 genes. In the significant top 20 KEGG pathways, three pathways were closely related to this study, namely, the cGMP-PKG signalling pathway (map04022), cAMP signalling pathway (map04024), and Hippo signalling pathway (map04390).

The eight genes enriched in the cGMP-PKG signalling pathway are: *CNGB1* is a nucleotide-gated channel 1, and its function is to respond to light-induced changes in intracellular cGMP levels^13^; the protein encoded by the *PPIF* gene is a peptide A member of the prolyl cis–trans isomerase (PPIase) family^14^; *ADRA1B* is the adrenergic receptor α-1B^15^; *GTF2I* is the transcription initiation factor; *AKT* is serine, threonine Acid kinase^16^; *KCNMB1* is the largest potassium channel, which has the characteristics of large conductance, large voltage, and calcium sensitivity, and is the basis for smooth muscle tone and neuronal excitability control^17^. *PPP1C* is the catalytic subunit of myosin light chain phosphatase (*MLCP*). It and myosin light chain kinase (*MLCK*) are phosphorylated and dephosphorylated so that myosin can activate myosin ATPase, thereby causing smooth muscle contraction activity^18,19^.

The eight genes enriched by the cAMP signalling pathway are as follows: *ADCY10* is adenylate cyclase 10, which regulates cAMP levels under the action of carbonate ions and calcium ions^20^; *CNGB1* is cyclic nucleotide-gated channel 1, which is used in calcium ions. Play a role in CAM under the action^21^. *PPP1C* is the catalytic subunit of serine/threonine protein phosphatase (PP1); *AKT* is a serine, threonine kinase; *HCN2* is cyclic nucleotide-gated channel 2 activated by potassium hyperpolarization^22^; and *PPP1R1B* is a protein phosphate enzyme 1 that regulates subunit 1B^23^. *PKA* has a regulatory effect on bile secretion, insulin secretion, and apical chloride channels.

Finally, five genes were enriched in the Hippo signalling pathway: the *BMP8* gene encodes the secretory ligand of transforming growth factor superfamily protein. Transforming growth factor is a multifunctional protein that can regulate the growth and differentiation of various cells, as well as apoptosis and cellular immunity^24^. Protein phosphatase 1 (*PP1*) has three catalytic subunits, one of which is the protein encoded by the PPP1C gene. PP1 is a serine/threonine-specific protein phosphatase that is acknowledged to participate in the regulation of all kinds of cellular processes, for instance muscle contractility, cell division, glycogen metabolism, and protein synthesis^25^. *YWHAH* gene products belong to the highly conserved 14-3-3 family of proteins, which are mediated by binding to phosphoserine proteins. Signal transduction inhibits cell division^26^. The *BIRC5* gene is a member of the inhibitor of apoptosis (IAP) gene family, and the IAP gene family encodes negative regulatory proteins that inhibit apoptotic cells^27^. The protein family encoded by the *FZD1* gene serves as a Wnt signal, and the transmembrane receptors of the pathway play a very important role in the growth and development of animals^28^.

## Discussion

In this study, the small intestine tissues of Asian Badger and Northern Hog Badger (three each) were used for high-throughput transcriptome sequencing, and high-quality offline transcriptome data were obtained. First, the quality of the sequencing data was evaluated, and then the error rate and data filtering were checked. Clean reads of 23G and 24.65G were obtained from Asian Badgers and Northern Hog Badgers, respectively. Then, the unigenes of the Asian Badger and Northern Hog Badger were annotated. From the percentage of the annotated unigenes to the total unigenes, the ratio of the annotations to the unigenes in the seven databases was not high. It is hypothesized that there are too many unigenes below 500 bp that are not easy to annotate. However, according to the number of unigene comments, 112,596 unigenes were annotated by Asian Badgers, and 131,381 unigenes were annotated by Northern Hog Badgers, which showed a good annotation effect. In the GO classification of unigenes, it was found that there were three GO items involved in digestion. In the KEGG classification, the digestive system subclass contains 695 and 611 unigenes in Asian Badgers and Northern Hog Badgers, respectively, involving 9 pathways. The results showed that the expression and annotation of genes related to digestion were very good in the small intestine of Asian Badgers and Northern Hog Badgers. The GO enrichment analysis of divergent orthologous genes can understand the biological processes involved, the molecular functions, and the cellular environment in which they are involved. The results of significant enrichment showed that eight terms in the molecular function category were related to six enzyme activities. This shows that the genes related to the activities of these six enzymes in the small intestines of Asian Badgers and Northern Hog Badgers are under positive selection pressure. The rapid evolution of these genes may affect the activity of corresponding enzymes, thus improving the digestive function of animals.

The small intestine is in a dynamic state of secretion and absorption, the sum of which results in net absorption^29^. There are two types of glands in the small intestine, duodenal gland and intestinal gland^30^. The main secretion of the duodenal glands is an alkaline liquid, which contains highly viscous mucin, and its main function is to protect the duodenal epithelium from gastric acid. The intestinal glands secrete small intestinal juice, which is composed of enterokinase and small intestinal amylase. Enterokinase can activate trypsinogen, and the role of trypsin is to activate various zymogens in the digestive system, so enterokinase has always been considered to be digestive one of the important starting enzymes of the system^31^. The secretion of the small intestine is mainly due to the activation of the second messengers cAMP, cGMP, and calcium to squeeze out chloride and bicarbonate through the apical chloride channel. The cGMP-PKG signalling pathway is an intracellular second messenger that plays a role through nitric oxide (NO) and diuretic natriuretic peptide (NPs) and regulates the increase in cGMP levels in many physiological processes, mainly through cGMP-dependent protein kinase (PKG). The cAMP signalling pathway, also known as the PKA system (protein kinase A system, *PKA*), is a cyclic nucleotide system. In this system, extracellular signals are combined with corresponding receptors to cause reactions by regulating the level of the second messenger cAMP in the cell. The signalling molecules are usually hormones, and the regulation of cAMP levels is carried out by adenylate cyclase. The cAMP and cGMP signalling pathways also affect the transport of substances in the small intestine^32,33^. In the KEGG enrichment analysis of divergent orthologous genes, there were eight genes in the cGMP-PKG signalling pathway, and six genes were enriched in the cAMP signalling pathway. The results suggest that these genes may have boosted the secretion and transport capacity of the small intestine of Asian Badgers and Northern Hog Badgers to aid digestion.

In mammals, the upstream membrane protein receptor of the Hippo signalling pathway acts as a sensor for extracellular growth inhibition signals. When it senses the extracellular growth inhibition signal, it activates a series of kinase cascade phosphorylation reactions and phosphorylation downstream effect factors YAZ and TAP. Phosphorylated YAP and TAZ bind to cytoskeletal proteins, causing them to stay in the cytoplasm and reduce their nuclear activity, thereby regulating organ volume and size^34–37^. The small intestine is the main organ for absorbing nutrients, which is related to its physiological structure. First, the small intestine has a huge absorption area, there are circular folds on the small intestinal mucosa, there are many villi on the folds, and there are microvilli on the epithelial cells of the villi. This structure increases the absorption area of the small intestine by approximately 600 times^30,38^. Second, the villi of the small intestine are rich in capillaries and lymphatic capillaries. Additionally, the mechanical movement of the small intestine and the activity of the villi can promote the return of lymph and blood and contribute to the absorption of nutrients^39^. In this study, six unigenes were enriched in the Hippo signalling pathway, and they were related to cell division, differentiation, apoptosis, and growth and development of organisms. These results and theories suggest that these rapidly evolving genes may influence the ability of the small intestines of Asian Badgers and Northern Hog Badgers to absorb nutrients.

Meanwhile, KEGG enrichment also involves cell adhesion molecules (CAMs) and ribosome biogenesis in eukaryotes. Cell adhesion molecules (CAMs) are (glyco) proteins expressed on the cell surface and play a critical role in a wide array of biological processes that include haemostasis, the immune response, inflammation, embryogenesis, and the development of neuronal tissue. Ribosomes are the cellular factories responsible for making proteins^40^. In eukaryotes, ribosome biogenesis involves the production and correct assembly of four rRNAs and approximately 80 ribosomal proteins. It requires hundreds of factors not present in the mature particle. In the absence of these proteins, ribosome biogenesis is stalled, and cell growth is terminated even under optimal growth conditions^41^. The result may have been an increase in their ability to fight inflammation and cell growth.

## Conclusions

Our study provided small intestinal transcriptome data from Asian Badgers and Northern Hog Badgers. We reveal the molecular basis of the small intestinal digestion and absorption of Asian Badgers and Northern Hog Badgers, members of the family supercarnivore (Mustelidae), which are omnivorous in their diet. Our analysis of the small intestine transcriptome of the Asian Badger and Northern Hog Badger can provide important data resources for the study of food habits and the digestion of Mustelidae. Additionally, it is worth noting that we are only looking at Asian Badgers and Northern Hog Badgers. However, there are many omnivorous animals, such as the Wolverine, the Honey Badger, and the American Badger. Therefore, it is necessary to further investigate whether the genetic signatures found in Asian Badgers and Northern Hog Badgers are also shared in other omnivores and/or if the other omnivores show different patterns of evolutionary adaptation according to their major food types.

## Methods

### Sample collection and RNA extraction

The collection of experimental animals was carried out according to the principle for animal experimentation. The experimental materials were the small intestine of three Asian Badgers and the small intestine tissue of three Northern Hog Badgers. The animals were all healthy adults. The animals were obtained from a Lelai Huan special farm (Laiwu District, Jinan, Shandong Province, China). All animals were raised under the same conditions with the same food. Laboratory animal were euthanized by carbon dioxide asphyxiation followed by decapitation according to the published protocol and the institutional guidelines on animal welfare. The small intestine was cut into pieces and placed in a 5 mL centrifuge tube (the centrifuge tube was treated with DEPC in advance to prevent RNase from degrading RNA), followed by adding an appropriate amount of RNA Later (RNA Stabilization Reagent, QIAGEN), and quickly stored in the refrigerator at -80℃. To distinguish the samples, the three Asian Badgers were named GH1, GH2, and GH3, and three Northern Hog Badgers were named ZH1, ZH2, and ZH3. Total RNA was extracted from the 6 small intestine samples using RNeasy MiNi Kit (QIAGEN, USA). The quality control of RNA samples mainly includes four methods. First, agarose gel electrophoresis was used to detect the degree of total RNA degradation and contamination. RNA purity was checked using a NanoPhotometer spectrophotometer (IMPLEN, CA, USA). RNA concentration was measured using a Qubit RNA Assay Kit in a Qubit 2.0 Flurometer (Life Technologies, CA, USA). RNA integrity was assessed using the RNA Nano 6000 Assay Kit of the Agilent Bioanalyzer 2100 system (Agilent Technologies, CA, USA).

### Library preparation and reference transcription assembly

Sequencing libraries were generated using the NEBNext Ultra RNA Library Prep Kit for Illumina (NEB, USA) following the manufacturer’s recommendations. After the sample was qualified, the mRNA was enriched with Oligo (dT) magnetic beads. The mRNA was fragmented by adding a fragmentation buffer. Using mRNA as a template, one strand of cDNA was synthesized with random hexamers. Then, two-strand cDNA was synthesized by adding buffer, dNTPs, DNA polymerase I and RNase H. Purified double-stranded cDNA was first repaired by adding a tail and connecting sequencing adapters. Then, AMPure XP Beads (Beckman Coulter, Beverly, USA) were used to select the fragment size. Finally, PCR amplification was performed, and to preferentially select cDNA fragments 150–200 bp in length, the library fragments were purified with the AMPure XP system. After the library was constructed, Qubit2.0 was used for preliminary quantification, and the library was diluted to 1.5 ng/µL. Agilent 2100 was used to detect the insert size of the library. After the insert size met the expectation, the qPCR method was used to accurately quantify the effective concentration of the library (effective concentration > 2 nM) to ensure library quality. After the library was qualified, the different libraries were pooled according to the effective concentration and the target off-machine data volume for Illumina HiSeq 2500 sequencing. The library preparations were sequenced on an Illumina HiSeq 2500 platform, and 250 bp paired-end reads were generated. Because the genomes of these two animals have not yet been published, the sequence obtained from sequencing can be spliced into a transcript, and the transcript can be used as the reference sequence for subsequent analysis. We used Trinity^42^ to assemble clean reads to obtain reference sequences. We took the longest transcript in each gene as a unigene to annotate gene functions.

### Unigene functional annotation

In annotating Unigene functionality, we used seven public databases (NCBI, NR, Swiss-Prot, PFAM, KEGG, GO, and KOG) through BLAST. Gene Ontology (GO) assignments were used to classify the functions of the predicted Asian Badger and Northern Hog Badger genes. Additionally, the unigenes were also classified according to Eukaryotic Orthologous Groups (KOG) terms.

### CDS forecasting and analysis of orthologous genes

To obtain an overview of the Asian Badger and Northern Hog Badger small intestine transcriptome, a cDNA sample was prepared from the small intestine of these two species, with three replicates for each species, and sequenced using the Illumina sequencing platform. To guarantee the quality of information analysis, raw reads must be filtered to obtain clean reads. We trimmed adapters, low-quality reads, and reads with a proportion of base information greater than 10% in raw reads to obtain clean reads for subsequent analysis. CDS (Coding sequence) forecasting is a two-step process. Unigenes were compared according to the priority sequence of the NR protein library and SwissProt protein library. If so, open reading frame (ORF) coding box information of the transcript was extracted from the comparison results, and the coding region sequence was translated into the amino acid sequence according to the standard codon table (5′-3′). Estscan (http://estscan.sourceforge.net/) software was used to predict the ORF of the NR protein library and SwissProt protein library that were not compared to obtain nucleic acid and amino acid sequences encoded by this part of the gene. We screened CDSs obtained by BLAST comparison and analysis, obtained full-length CDSs, and performed UTR prediction. These full-length CDSs are more likely to be direct orthologous sequences^43^.

### Analysis of orthologous genes ka/ks

First, we used OrthoMCL^44^ to perform an orthologous gene search on full-length CDSs and screened one-to-one orthologous genes. We took ferret as the internal reference and giant panda as the external reference according to kinship. Afterwards, Muscle^44^ was used to compare and analyse the protein sequences of orthologous genes and optimize the results of protein sequence alignment through Gblocks (http://www.vardb.org/vardb/analysis/gblocks.html) to eliminate the sites that were poorly aligned or aligned to multiple regions and converted into nucleic acid sequence alignment results. Self-to-self BLASTP was conducted for all amino acid sequences with a cut-off E-value of 1e−5. Orthologous groups were constructed from the results with OrthoMCL using default settings. We used paml-codeml^45^ to calculate which of these orthologous genes belong to synonymous substitutions and which belong to nonsynonymous substitutions and calculate Ka/Ks. During the analysis, we calculated the proportion of synonymous and nonsynonymous mutations by treating the orthologous genes as a single gene. In genetics, Ka/Ks or dN/dS represents the ratio between the nonsynonymous substitution rate (Ka) and the synonymous substitution rate (Ks). This ratio can determine whether there is selective pressure on this protein-coding gene. If Ka/Ks > 1, it is considered that there is a positive selection effect; if Ka/Ks = 1, it is considered that there is neutral selection; and if Ka/Ks < 1, it is considered that there is a purification selection effect^45^. When Ka/Ks > > 1, there are many nonsynonymous mutations in the sequence, which are strongly positively selected. Such genes are genes that have recently rapidly evolved (divergent) and have very important significance for the evolution of species. This means that “divergent orthologous genes” were conserved between Asian Badger and Northern Hog Badger but divergent when compared to other species. In contrast, when Ka/Ks < 0.1 or closer to 0, mainly synonymous mutations occur in the sequence. These genes are relatively conserved and are evolutionarily subject to strong selection constraints.

### GO and KEGG pathway enrichment analyses

Gene Ontology (GO, http://www.geneontology.org/) is an international standard classification system for gene function. GO enrichment analysis method was GOseq^46^. This method was based on a Wallenius noncentral hyperdistribution.

KEGG (Kyoto Encyclopedia of Genes and Genomes) is the main public database for pathways^47^. The KEGG pathway was used as the unit of significance enrichment analysis, and the supergeometric test was applied to identify the channels with significant enrichment of the divergent and conserved family homologous genes relative to all the annotated genes. We used Kobas^48^ software to perform KEGG pathway enrichment analysis on divergent (Ka/Ks > 1) and conserved (Ka/K < 0.1) orthologous gene sets.

### Ethics statement

All sample procedures and experimental methods were approved by the Qufu Normal University Institutional Animal Care and Use Committee (No. QFNU2017–019), Qufu, China.

## Supplementary Information

Supplementary Information 1.

Supplementary Information 2.

Supplementary Information 3.

## Data Availability

All data are available at the NCBI: Asian Badger: PRJNA675668 Northern Hog Badger: PRJNA675731.
